# Pathways of Carbon and Energy Metabolism of the Epibiotic Community Associated with the Deep-Sea Hydrothermal Vent Shrimp *Rimicaris exoculata*

**DOI:** 10.1371/journal.pone.0016018

**Published:** 2011-01-07

**Authors:** Michael Hügler, Jillian M. Petersen, Nicole Dubilier, Johannes F. Imhoff, Stefan M. Sievert

**Affiliations:** 1 Biology Department, Woods Hole Oceanographic Institution, Woods Hole, Massachusetts, United States of America; 2 Leibniz Institute of Marine Sciences, IFM- GEOMAR, Kiel, Germany; 3 Max Planck Institute for Marine Microbiology, Bremen, Germany; Max-Planck-Institute for Terrestrial Microbiology, Germany

## Abstract

**Background:**

The shrimp *Rimicaris exoculata* dominates the faunal biomass at many deep-sea hydrothermal vent sites at the Mid-Atlantic Ridge. In its enlarged gill chamber it harbors a specialized epibiotic bacterial community for which a nutritional role has been proposed.

**Methodology/Principal Findings:**

We analyzed specimens from the Snake Pit hydrothermal vent field on the Mid-Atlantic Ridge by complementing a 16S rRNA gene survey with the analysis of genes involved in carbon, sulfur and hydrogen metabolism. In addition to Epsilon- and Gammaproteobacteria, the epibiotic community unexpectedly also consists of Deltaproteobacteria of a single phylotype, closely related to the genus *Desulfocapsa*. The association of these phylogenetic groups with the shrimp was confirmed by fluorescence in situ hybridization. Based on functional gene analyses, we hypothesize that the Gamma- and Epsilonproteobacteria are capable of autotrophic growth by oxidizing reduced sulfur compounds, and that the Deltaproteobacteria are also involved in sulfur metabolism. In addition, the detection of proteobacterial hydrogenases indicates the potential for hydrogen oxidation in these communities. Interestingly, the frequency of these phylotypes in 16S rRNA gene clone libraries from the mouthparts differ from that of the inner lining of the gill chamber, indicating potential functional compartmentalization.

**Conclusions:**

Our data show the specific association of autotrophic bacteria with *Rimicaris exoculata* from the Snake Pit hydrothermal vent field, and suggest that autotrophic carbon fixation is contributing to the productivity of the epibiotic community with the reductive tricarboxylic acid cycle as one important carbon fixation pathway. This has not been considered in previous studies of carbon fixation and stable carbon isotope composition of the shrimp and its epibionts. Furthermore, the co-occurrence of sulfur-oxidizing and sulfur-reducing epibionts raises the possibility that both may be involved in the syntrophic exchange of sulfur compounds, which could increase the overall efficiency of this epibiotic community.

## Introduction

Highly specific epibiotic associations between bacteria and invertebrates are a very common phenomenon at deep-sea hydrothermal vents, contributing to overall biomass production at these sites [1] (and references therein). The caridean shrimp *Rimicaris exoculata* [2] is well known for its association with epibiotic bacteria. These shrimp dominate the faunal biomass at many deep-sea hydrothermal vent sites on the Mid-Atlantic Ridge (MAR), where they form large swarms around active sulfide structures, reaching abundances up to 3,000 individuals per m^2^ [3] (and references therein). *R. exoculata* harbors specialized epibiotic bacterial communities on the inner lining of the wall of its expanded branchial chamber, the so-called branchiostegites, and on its modified mouthparts, the scaphognathites and expodites of the first maxillipeds [4]–[6]. A number of studies have focused on the nature of this association and its benefits for the shrimp. The chemoautotrophic potential of the community has been confirmed by activity of ribulose 1,5-bisphosphate carboxylase/oxygenase (RubisCO) and incorporation of radioactively labeled bicarbonate into biomass [7]–[10]. Furthermore, bulk stable carbon and nitrogen isotopic studies as well as compound specific stable carbon isotope analyses clearly indicated that at least some of the epibionts are chemoautotrophic, and the shrimp's biomass also has stable isotopic signatures typical of animals that rely on chemoautotrophic bacteria for their nutrition [10]–[13]. This is suggestive of a nutritional role of the epibionts for the shrimp. However, the transfer mechanism of organic matter from the epibionts to the shrimp remains elusive [3]. In addition to the epibionts residing in the branchial chamber, a resident gut microbial community might also contribute to the nutrition of the shrimp [10], [14], [15], as well as sulfide-associated microbes ingested by the shrimp [4].

It has been proposed that the shrimp aggregate in the mixing zone of hydrothermal fluids and ambient deep-sea water to supply the epibionts with both oxidants and reductants to drive chemoautotrophy [16]. However, the actual energy sources used by the epibionts remain unclear. Sulfide oxidation was initially seen as the most likely metabolism [8], [9], [11], [17]. This was concluded from the observation of sulfur globules in epibiont filaments [11], the phylogeny of the epibionts [17], and the fluid chemistry of the initially studied vent sites, i.e., TAG and Snake Pit, both of which are characterized by high sulfide concentrations [18]. However, the presence of large aggregations of *R. exoculata* shrimp at vent sites where the hydrothermal fluids contain high iron, methane, and hydrogen concentrations, and relatively low sulfide concentrations, like for example the Rainbow vent site [19], led to the proposal that alternative energy sources, notably the oxidation of ferrous iron, could drive chemoautotrophy of the epibionts [20], [21]. Indeed, thermodynamic modeling predicted that sulfide oxidation would be the predominant energy source at the TAG vent site, whereas at the Rainbow vent site most energy could be gained by the oxidation of the abundant iron and hydrogen and possibly methane [16]. However, the role of biologically mediated iron oxidation has recently been challenged [22].

In a pioneering study, Polz and Cavanaugh [17] showed that the *R. exoculata* epibiotic community from the Snake Pit vent site was composed exclusively of one phylotype belonging to the Epsilonproteobacteria [17]. However, more recently the epibiotic microbial community of shrimp collected at other sites on the MAR (Rainbow, TAG, Logatchev, South MAR) has been investigated in more detail, providing evidence for the presence of a more phylogenetically and functionally diverse epibiotic community [23], [24]. Differences in the epibiotic communities at different hydrothermal vent sites described in previous studies could be due to the contrasting chemistries and thus potential availability of different electron donors at the different sites. Alternatively a similarly diverse epibiont community might also be present on shrimp from the Snake Pit vent site. To address this question and to obtain information about the potential energy source(s) that might drive the chemoautotrophic metabolism of the epibiotic community, we analyzed four *R*. *exoculata* individuals from the Snake Pit hydrothermal field by combining a 16S rRNA gene based diversity assessment with a survey of metabolic genes involved in carbon, sulfur, and hydrogen metabolism. In addition, fluorescence in situ hybridization was performed to verify the presence of the detected phylotypes and their location.

## Results

### Phylogenetic diversity of epibionts

**Figure 1:**
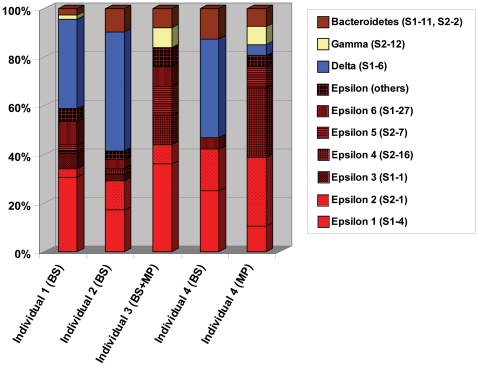
Relative proportions of the 16S rRNA gene defined OTUs in the clone libraries of different individuals and of different body parts of individual 4. See Table 1 for the actual number of sequences per OTU. BS  =  Branchiostegites, MP  =  Mouthparts.

To determine the phylogenetic diversity of the bacteria residing inside the branchial chamber of *R. exoculata,* 16S rRNA gene clone libraries were constructed from four different individuals. Two clone libraries originated from DNA extracted from the branchiostegites of individuals 1 (113 clones) and 2 (82 clones), respectively. A third clone library was constructed from the DNA extracted from both the branchiostegites and mouthparts of individual 3 (25 clones), while the fourth and fifth libraries were constructed with DNA extracted from the mouthparts (67 clones) and branchiostegites (64 clones) of individual 4, respectively (Table 1). This approach allowed us to assess the variability among the *R. exoculata* individuals analyzed, as well as the heterogeneity within the branchial chamber of a single individual. Analyses of 16S rRNA gene clone libraries revealed a diverse community that was composed of Epsilon-, Delta-, and Gammaproteobacteria as well as *Bacteroidetes*. While the Epsilonproteobacteria constituted a dominant fraction of the clones on both branchiostegites and mouthparts, the Delta- and Gammaproteobacteria were mainly detected in the clone libraries from the branchiostegites and mouthparts (Table 1 and Figure 1). *Bacteroidetes* were a minor, but consistent component of the clone libraries from all individuals and also on both body parts (Table 1 and Figure 1). The overall clone library composition was largely consistent among the analyzed individuals, although no Deltaproteobacteria were found in the clone library from individual 3.

**Table 1:** 16S rRNA gene clone library results.

Individual No.	1	2	3	4	4
Sampled compartment	BS	BS	BS+MP	BS	MP
Epsilon S1-4	35	14	9	16	7
Epsilon S2-1	4	10	2	11	19
Epsilon S1-1	7	2	0	0	0
Epsilon S2-16	1	2	3	0	19
Epsilon S2-7	3	0	3	0	6
Epsilon S1-27	11	3	2	3	0
Epsilon Others	6	3	2	0	3
Gamma S2-12	1	0	2	0	5
Delta S1-6	42	40	0	26	3
*Bacteroidetes* S1-11	3	4	0	4	1
*Bacteroidetes* S2-2	0	4	2	4	4
Total No. of sequences	113	82	25	64	67

BS  =  Branchiostegites, MP  =  Mouthparts.

**Figure 2:**
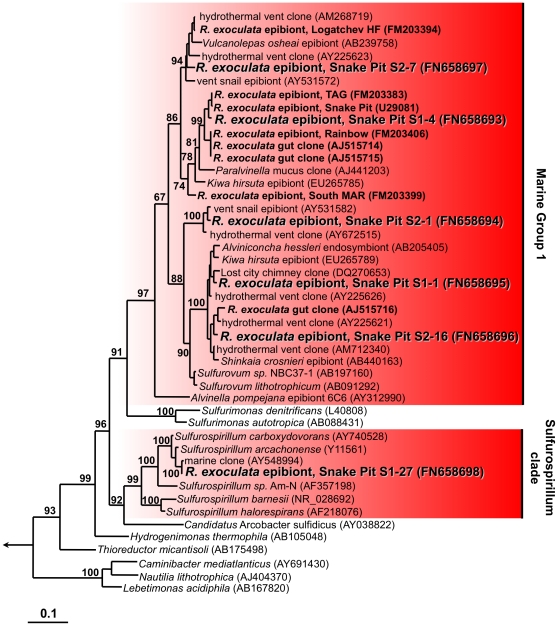
16S rRNA gene based phylogeny of Epsilonproteobacteria. The tree was calculated using the Maximum-Likelihood method. Bootstrap values are shown as percentages of 100 bootstrap replicates. Sequences obtained in the present study are depicted in color. Scale bars represent 10% estimated sequence divergence.

The epsilonproteobacterial community was quite diverse, with five OTUs (S2-7, S1-4, S2-16, S1-1, S2-1), representing 49% of the total recovered sequences, belonging to the Marine Group 1 (MG1) cluster within the *Thiovulgaceae* [25] and one OTU (S1-27), representing 4% of all sequences, belonging to the *Sulfurospirillum* cluster (Table 1, Figure 1 and Figure 2). The dominant epsilonproteobacterial OTU (S1-4) was most closely related to the phylotype originally described from *R. exoculata* from Snake Pit [17] (Figure 2). The other OTUs showed highest similarities to clones from either animal-associated or free-living microbes from other hydrothermal vent sites [14], [26]–[32] (Figure 2).

**Figure 3:**
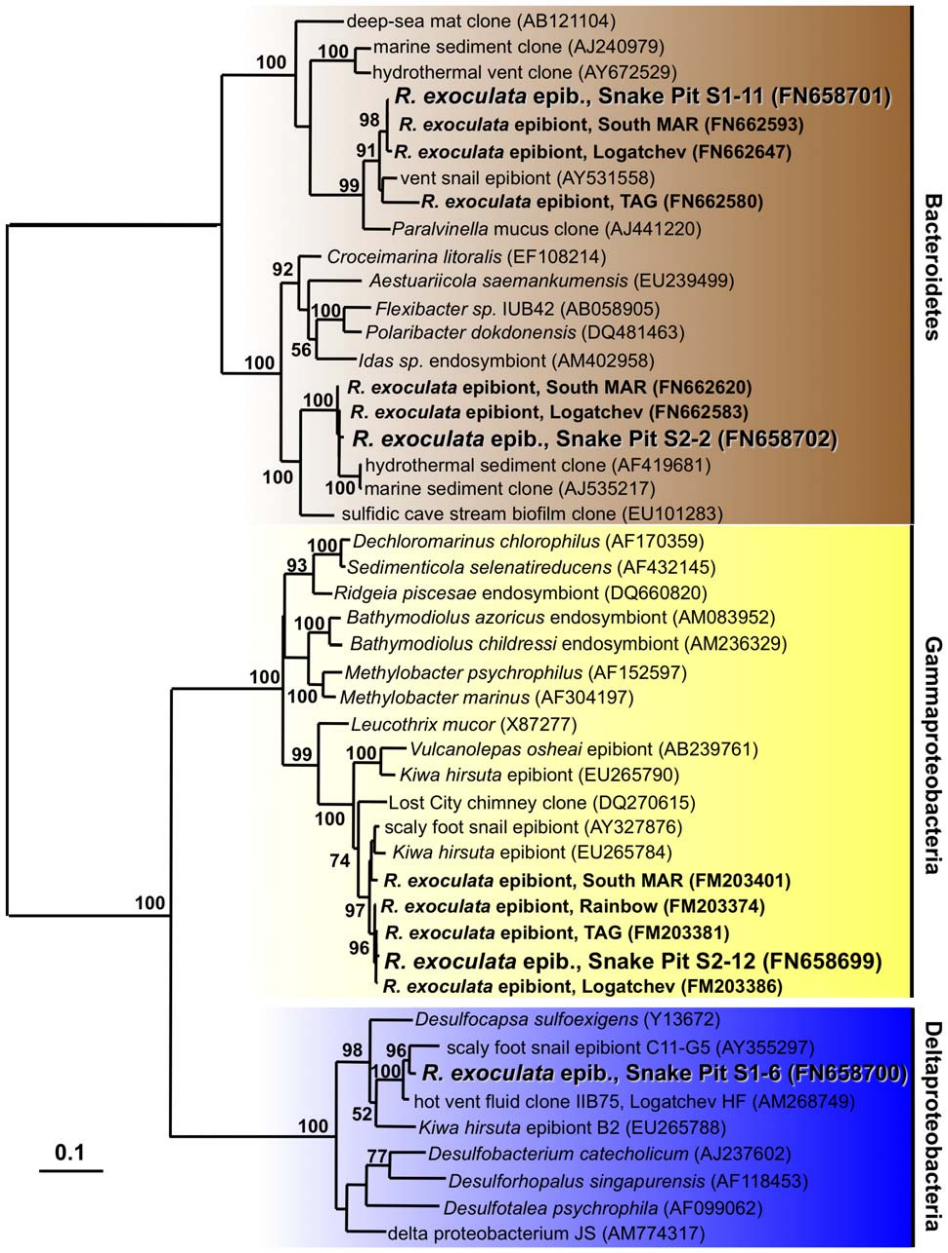
16S rRNA gene based phylogeny of Gamma- and Deltaproteobacteria and *Bacteroidetes*. The tree was calculated using the Maximum-Likelihood method. Bootstrap values are shown as percentages of 100 bootstrap replicates. Sequences obtained in the present study are depicted in color. Scale bars represent 10% estimated sequence divergence.

Deltaproteobacteria constituted a large fraction of the observed clones (32%), in particular on the branchiostegites (Table 1 and Figure 1). All 111 sequences retrieved from 3 individuals represented a single OTU (OTU S1-6) with the closest cultivated relatives being *Desulfocapsa sulfoexigens* and *Desulfocapsa thiozymogenes*, bacteria capable of disproportionating partially oxidized sulfur compounds [33], [34] (Figure 3). Closely related uncultivated bacteria included sequences retrieved from other hydrothermal microbe-animal associations [27], [29].

Gammaproteobacteria constituted a small, but consistent fraction of the recovered clones (Table 1 and Figure 1). Only one OTU was detected that was most closely related to clones recently shown to be associated with *R. exoculata* from four vent sites on the MAR [24] (Figure 3). The closest, albeit distantly related, cultivated relative is *Leucothrix mucor*, a chemolithoheterotrophic sulfur oxidizer [35]. The OTUs affiliated with *Bacteroidetes* were most similar to sequences previously recovered from *R. exoculata* from three vent sites on the MAR [24] (Figure 3).

**Figure 4:**
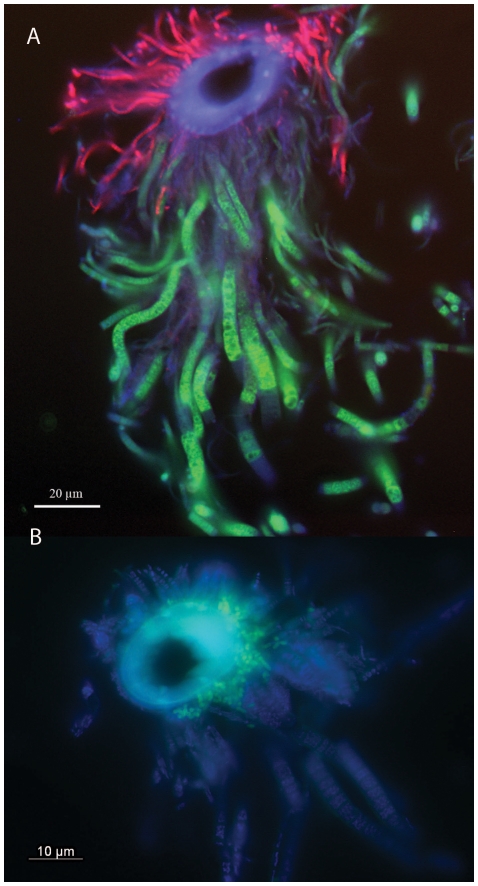
Fluorescence in situ hybridization (FISH) of sections through scaphognathite setae of a *Rimicaris exoculata* individual from Snake Pit. (A) FISH showing the gammaproteobacterial symbiont in red, hybridized with the probe Rexogam1268RT, and the epsilonproteobacterial epibiont in green, hybridized with the probe RexoepsTG996 (400x). (B) FISH showing deltaproteobacterial epibiont in green, hybridized with probe SRB385 (1000x). In both cases, DAPI stained cells are blue. The images were taken with a Zeiss Axioskop II epifluorescence microscope.

Fluorescence in situ hybridization (FISH) of sections through the mouthparts of an *R. exoculata* individual from Snake Pit generally reflected the results of the 16S rRNA gene clone libraries from the mouthparts (Figure 4). A specific probe from Petersen *et al.* [24], which targets the epsilonproteobacterial epibiont OTU S1-4, stained large filaments (width approximately 3 µm), while a gammaproteobacterial epibiont probe from Petersen *et al.* [24] hybridized with thinner filaments (width approximately 1 µm) (Figure 4A). In addition, the general deltaproteobacterial probe SRB 385 [36] stained small cocci at the base of the setae (Figure 4B).

### Carbon fixation

To assess the autotrophic potential and the biosynthetic pathways involved in carbon fixation of the epibiotic community we constructed clone libraries for genes encoding for subunits of key enzymes of two carbon fixation pathways, RubisCO for the Calvin-Benson-Bassham (CBB) cycle and the ATP-dependent citrate lyase (ACL) for the reductive tricarboxylic acid (rTCA) cycle. The CBB cycle is known to be used for carbon fixation by a number of autotrophs including gammaproteobacterial chemoautotrophs [37], [38], whereas the rTCA cycle has been identified as the carbon fixation pathway used by all chemoautotrophic Epsilonproteobacteria investigated to date [39]–[41]. We therefore considered these the most likely pathways to be used for carbon fixation by the gamma- and epsilonproteobacterial epibionts. We used primers that target fragments of the genes encoding for the large subunits of RubisCO form I (*cbbL*) and II (*cbbM*), as well as primers that target fragments of the small (*aclB*) and large (*aclA*) subunits of ACL.

**Figure 5:**
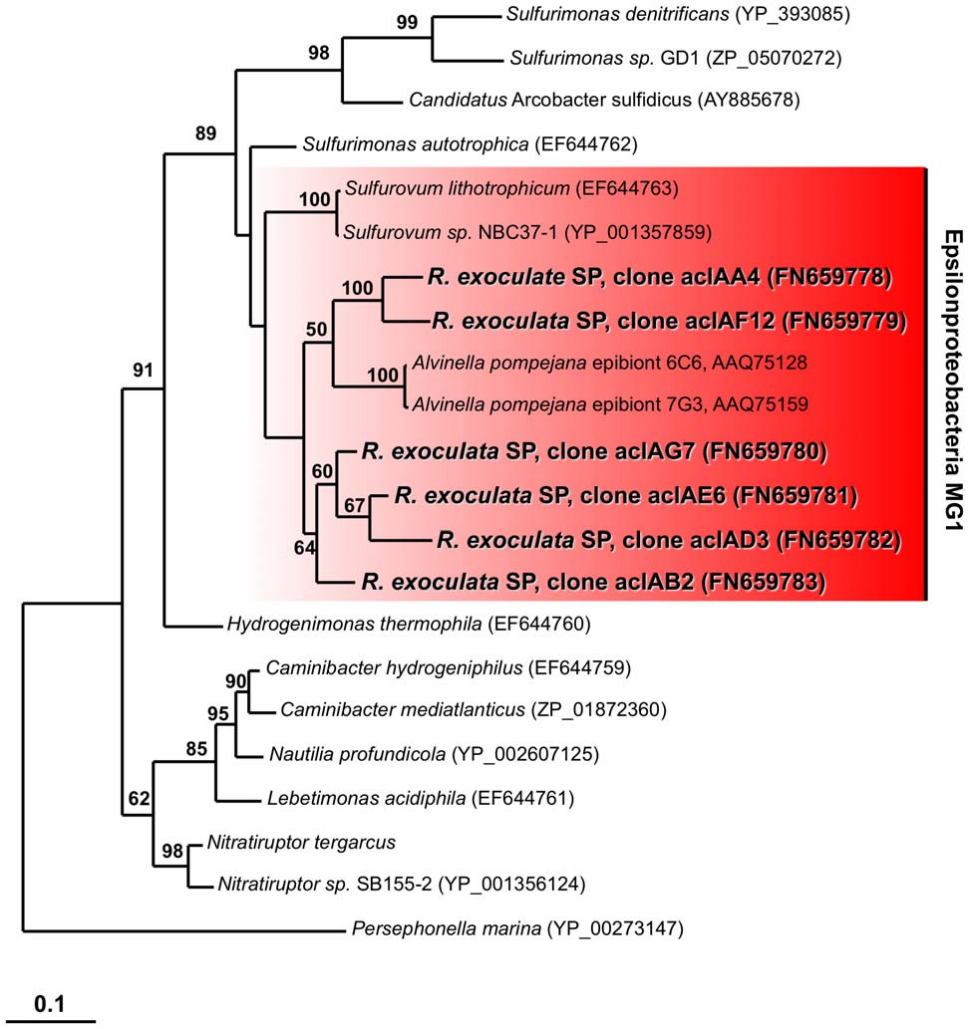
Phylogenetic tree based on translated *aclA* sequences. The tree was calculated using the Maximum-Likelihood method. Bootstrap values are shown as percentages of 100 bootstrap replicates. Sequences obtained in the present study are depicted in red. Scale bar represents 10% estimated sequence divergence.

As expected, fragments of both *aclA* and *aclB* coding for the two subunits of ATP citrate lyase were detected in the clone libraries from both the mouthparts and the branchiostegites. All *acl* sequences belonged to a clade containing only sequences from Epsilonproteobacteria (Figure 5 and Figure S1). Consistent with the 16S rRNA gene data for the epsilonproteobacterial community, the *acl* sequence analyses revealed several sequence types, and most *aclA* and *aclB* sequences formed a cluster with *acl* sequences from *Sulfurovum* species of the MG1 clade within the *Thiovulgaceaea*. The gene *cbbM* coding for RubisCO form II could only be amplified from the mouthparts (Table S1), corresponding to the presence of Gammaproteobacteria in our 16S rRNA gene clone libraries. Only one sequence type was retrieved that clustered with *cbbM* sequences from chemautotrophic Gammaproteobacteria of the genus *Thiomicrospira*, and it was most closely related to the *cbbM* gene of an uncultured bacterium from the Logatchev vent field on the MAR (Figure S2). No *cbbL* genes coding for RubisCO form I could be amplified, which is in line with a previous study that based on immunoblotting found evidence for the presence only of RubisCO form II [42].

### Energy metabolism

**Figure 6:**
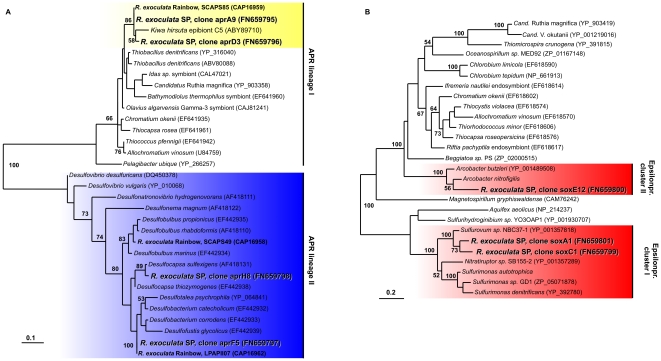
Phylogenetic trees based on translated *aprA* (A) and *soxB* (B) sequences. The trees were calculated using the Maximum-Likelihood method. Bootstrap values are shown as percentages of 100 bootstrap replicates. Sequences obtained in the present study are shown in color. Scale bars represent 10% (A) and 20% (B) estimated sequence divergence, respectively.

To determine the potential energy-generating pathways for chemoautotrophic carbon fixation, we amplified fragments of genes coding for subunits of key enzymes involved in the oxidation or reduction of sulfur compounds as well as hydrogen oxidation. A fragment of the gene coding for 5′-adenylylsulfate reductase (*aprA*), a key gene for the oxidation of reduced sulfur compounds and the reduction of sulfate, could be amplified. *AprA* sequences related to sequences from gammaproteobacterial sulfur oxidizers were mainly amplified from the mouthparts, whereas sequences related to those of deltaproteobacterial sulfate-reducers, were almost exclusively amplified from the branchiostegites (Figure 6A and Table S1). These results are consistent with the distribution of these phylogenetic groups based on 16S rRNA gene clone libraries. Phylogenetic analyses of the *aprA* sequences revealed two closely related (97.7% amino acid sequence identity) sequence types related to gammaproteobacterial *aprA* sequences. These clustered together with clone SCAPS85 of an *R. exoculata* specimen from Rainbow and clone C5 from the Yeti crab *Kiwa hirsuta* [23], [29]. The deltaproteobacterial sequences fell into two separate clusters (Figure 6A). The most frequently retrieved sequence formed a cluster with sequences from the sulfur disproportionating species *Desulfocapsa sulfoexigens* and *Desulfocapsa thiozymogenes* (Fig. 6A). The second sequence type was only found once and was most closely related to a clone from *R. exoculata* at Rainbow [23].

A fragment of the gene coding for SoxB, a sulfate thiohydrolase that constitutes a key component of the Sox multienzyme complex involved in oxidation of reduced sulfur compounds [43], was amplified from both mouthparts and branchiostegites (Table S1). The sequences formed two *soxB* clusters, both of which were affiliated with Epsilonproteobacteria (Figure 6B).

In addition, we could amplify a portion of *hynL* encoding the large subunit of an [NiFe] hydrogenase, which is involved in the oxidation of molecular hydrogen for energy generation. The *hynL* sequences fell in three clusters, two in a group containing epsilonproteobacterial sequences and one in a group containing deltaproteobacterial sequences (Figure S3).

## Discussion

### Phylogenetic diversity and spatial heterogeneity

Analyses of 16S rRNA gene clone libraries of the epibiotic community of four *R. exoculata* individuals from Snake Pit revealed a diverse community that was composed of Epsilon-, Delta-, and Gammaproteobacteria, as well as *Bacteroidetes*. This is in contrast to the initial report that described the epibiotic community of *R. exoculata* from Snake Pit as a monoculture of one epsilonproteobacterial phylotype [17]. However, with the exception of the mouthparts of individual 4, the dominant epsilonproteobacterial phylotype in the clone libraries from all individuals of the present study (OUT S1-4) was most closely related to the phylotype originally identified by Polz and Cavanaugh [17]. The presence of various epsilonproteobacterial phylotypes in the epibiotic community has been recently reported for shrimp from other Mid-Atlantic Ridge vent fields [23], [24]. A total of 7 epsilonproteobacterial clades were identified from *R. exoculata* collected at Rainbow [23], although no information on the location of these bacteria is available because FISH analyses were not carried out. Petersen *et al.* [24] described the presence of only one, but phylogenetically distinct group of Epsilonproteobacteria (with less than 1% sequence difference) on *R. exoculata* at each of three vent fields along the MAR, with only those from Rainbow found to harbor two co-occurring groups of Epsilonproteobacteria (with 6% sequence difference), which was further confirmed by FISH. In the present study, we were able to confirm the dominance of OTU S1-4 on the scaphognathites by using a FISH probe specifically targeting this phylotype, but not the other epsilonproteobacterial sequences found in our clone libraries (Figure 4). However, given that four out of the six dominant epsilonproteobacterial OTUs were found in 16S rRNA clone libraries from all four individuals (Table 1 and Figure 1) it is likely that also these types might be a part of the epibiotic community, rather than being casually associated contaminants on the investigated shrimp. Indeed, according to our observations, not all filaments on the mouthparts hybridized to the probes used for FISH.

Besides Epsilonproteobacteria, we also identified Gamma- and Deltaproteobacteria as well as *Bacteroidetes* in the clone libraries of different individuals. The presence of Gamma- and Deltaproteobacteria was further confirmed by FISH, indicating that they are indeed part of the epibiotic community. In line with the biogeographical model developed by Petersen *et al.* [24], the 16S rRNA gene sequence of the gammaproteobacterial symbiont from Snake Pit falls within the cluster of sequences obtained from the northern MAR vent fields Rainbow, TAG, and Logatchev. The recovery of one deltaproteobacterial phylotype in high frequency from the clone libraries strongly suggests that it might be a significant component of the epibiotic community, in particular on the branchiostegites, with potential implications for sulfur cycling within the epibiotic community (see below). Sequences closely related to the *Bacteroidetes* phyloypes identified in the present study have been previously detected in shrimp from three other vent sites on the MAR [24] (Figure 3), but further work is needed to evaluate if they are indeed a an integral part of the epibiotic community.

In contrast to the Epsilonproteobacteria and *Bacteroidetes*, which were found on both the mouthparts and the branchiostegites, the gammaproteobacterial phylotype was only found in the clone library from the mouthparts and the deltaproteobacterial phylotype was mostly confined to the clone library from the branchiostegites. Furthermore, based on the results from the 16S rRNA gene clone libraries, we also found differences in the distribution of Epsilonproteobacteria between the branchiostegites and mouthparts. If this reflects differences in the abundance of these phylotypes on the shrimp, then this could be an indication for functional compartmentalization in different parts of the branchial chamber, which was originally proposed by Zbinden and colleagues [20].

### Carbon and energy metabolism

Based on the phylogenetic analyses of functional genes involved in energy metabolism and autotrophy, we are able to assign possible functions to the different epibiotic phylotypes. Due to the presence of *aprA* and *cbbM* sequences that group with sequences of gammaproteobacterial origin, and due to the fact that only one gammaproteobacterial phylotype was found in the 16S rRNA gene libraries, we consider it likely that the gammaproteobacterial epibiont has the potential to grow as a chemoautotrophic sulfur oxidizer, using the pathway involving Apr/Dsr for energy generation through the oxidation of reduced sulfur compounds and the CBB cycle for carbon fixation. The presence of two closely related *aprA* sequences could either be due to the presence of two copies of this gene in the genome of the gammaproteobacterial epibiont, which is known for other sulfur-oxidizing bacteria [44], or could represent variation within the gammaproteobacterial epibiont population that is not reflected in variation in the 16S rRNA gene. Considering that the closest cultured relative *Leucothrix mucor* is a chemolithoheterotroph [35], it is possible that the gammaproteobacterial epibiont of *R. exoculata* could be capable of mixotrophic growth, but this has not yet been investigated. Such a flexible metabolic strategy has been proposed for the sulfur-oxidizing symbiont of the giant tubeworm *Riftia pachyptila*, and may be more widespread among chemosynthetic symbionts than previously thought [45].

The phylogenetic grouping of *soxB* and *aclA/B* detected in this study with sequences from Epsilonproteobacteria, together with information available on cultivated representatives, suggests that the Epsilonproteobacteria found in association with *R. exoculata* use the Sox pathway for energy generation by oxidizing reduced sulfur compounds and the rTCA cycle for carbon fixation [39]–[41], [46]–[48]. In addition, hydrogen oxidation could also play a role, as indicated by the detection of *hynL* genes related to those of known Epsilonproteobacteria.

Besides Epsilonproteobacteria, the community on the branchiostegites also consisted of Deltaproteobacteria. Although they were mainly found in the clone libraries from the branchiostegites, FISH confirmed that they are also present on the mouthparts. Given their close phylogenetic relationship to bacteria of the genus *Desulfocapsa* and the presence of an *aprA* sequence related to *aprA* of *Desulfocapsa* spp., it is likely that the deltaproteobacterial phylotype we found on the shrimp has the gene for sulfite reduction, indicating genetic potential for sulfate reduction or the disproportionation of partially oxidized sulfur compounds. In addition, the presence of a hydrogenase related to those of deltaproteobacterial origin indicates that this bacterium could potentially grow lithotrophically using hydrogen as an electron donor for sulfate and/or sulfur reduction. Interestingly, Zbinden *et al.* [23] also detected 16S rRNA and *aprA* gene sequences most closely related to *Desulfocapsa* in low frequencies in clone libraries constructed from the epibiotic community of *R. exoculata* from the Rainbow vent site, indicating a potentially wider distribution of these bacteria. We hypothesize that an internal sulfur cycle between sulfur-oxidizing Epsilon- and Gammaproteobacteria and sulfate-reducing or sulfur-disproportionating Deltaproteobacteria could take place within the branchial chamber, similar to what has been proposed for an oligochaete symbiosis [49]. Such an internal sulfur cycle could possibly increase the overall efficiency of the epibiotic community. Syntrophic associations based on the exchange of reduced and oxidized sulfur compounds have also been described for a laboratory co-culture between a sulfur-oxidizing and a sulfate-reducing bacterium [50], aggregations between a (phototrophic) sulfur-oxidizer and a sulfate-reducer/sulfur disproportionator living in the chemocline of a meromictic lake [51], and the classical example of the sulfur reducer *Desulfuromonas acetoxidans* and a phototrophic green sulfur bacterium as sulfur oxidizer [52]. In all of these laboratory co-cultures, the growth efficiencies of both partners increased significantly in comparison to their growth in isolation.

The dominance of epsilonproteobacterial phylotypes recovered in the clone libraries and their abundance on the mouthparts as shown by FISH analyses suggests that the rTCA cycle could be contributing significantly to carbon fixation in the epibiotic community. In line with the multiple 16S rRNA-defined epsilonproteobacterial phylotypes, we observed a high diversity of *acl* sequences indicating that there may be multiple epsilonproteobacterial epibiont phylotypes that have the capacity to grow autotrophically. The use of the rTCA cycle for carbon fixation by the majority of the autotrophs would provide an alternative explanation for the previously reported heavy stable carbon isotope composition of the shrimp and epibionts, which is typically around −12‰ [4], [10]–[13]. This value is similar to the hydrothermal vent gastropod *Alviniconcha* aff. *hessleri* that contains epsilonproteobacterial endosymbionts using the rTCA cycle for carbon fixation [40], but outside the range typically associated with the CBB cycle [53], [54], that has previously been implied to be solely responsible for the observed stable carbon isotope composition of the epibionts and the shrimp [10], [12], [13]. Surprisingly, previously reported carbon fixation rates of the epibionts in the branchial chamber were comparatively low [9], [10], although our analysis as well as those of a previous study show the genetic potential for autotrophic growth by multiple members of the epibiotic community [23]. Further studies will be needed to address this discrepancy by simultaneously measuring carbon fixation rates under environmentally relevant conditions. For example, previous measurements were not performed at *in situ* pressure nor were precautions taken to prevent exposure to oxygen. The latter appears relevant if enzymes of the rTCA cycle and RubisCO form II are indeed the major catalysts of carbon fixation by the epibionts, as these are known to be oxygen-sensitive [25], [39], [55], [56]. Furthermore, determining the activities of key enzymes, as well as determining the relative contribution of the CBB and the rTCA cycle for overall carbon production are priorities for future research.

### Conclusions

This study describes the diversity and function of the epibiotic community in the branchial chamber of the hydrothermal shrimp *R. exoculata* from the Snake Pit hydrothermal vent site. The phylogeny of the epibionts based on the 16S rRNA gene was in most cases congruent with functional gene phylogenies, allowing us to develop hypotheses on the functions of the phylotypes associated with the shrimp. Epsilonproteobacteria could be performing sulfur oxidation via the Sox pathway and possibly hydrogen oxidation to drive carbon fixation via the rTCA cycle. The Gammaproteobacteria could be generating energy by oxidizing reduced sulfur compounds via the pathway involving Apr and Dsr to fix carbon via the CBB cycle. Similar epsilon- and gammaproteobacterial phylotypes have been identified in a number of epibiotic associations with various hydrothermal invertebrates (reviewed in [1]), in which they may perform similar functions. In addition, our data suggest the possibility of an internal sulfur cycle, in particular on the branchiostegites, driven by the syntrophic relationship between sulfur oxidizers and Deltaproteobacteria that perform the reduction of sulfate or disproportionation of partially oxidized sulfur species, such as elemental sulfur or thiosulfate. Given the small number of individuals available for this study as well as the limitations in appropriately preserving the samples for other types of analyses, it is clear that our results only provide a first assessment of the epibionts functional diversity. Further studies are needed to assess 1) the activity and relative importance of the different pathways, e.g., by combining FISH analyses of 16S rRNA and mRNA to link the different symbionts to specific metabolic pathways of energy and carbon fixation, and 2) the rates of carbon fixation under more realistic conditions, as well as to demonstrate the impact of environmental conditions on the distribution and abundance of different phylotypes associated with *R. exoculata*.

## Materials and Methods

### Sampling site, sample collection and processing

*R*. *exoculata* specimens from the Snake Pit hydrothermal vent site (23°22′N, 44°57′W, depth: 3600 m) were retrieved during cruise AMK-47 of the R/V 'Akademik Mstislav Keldysh' in 2002. The geology and geochemistry of the site has been described previously [18], [57], [58]. Upon recovery, shrimp were immediately frozen at −80°C for subsequent nucleic acid extraction. In addition, the mouthparts of some shrimp were prepared, fixed in ethanol, and stored at −20°C for subsequent analyses by fluorescence in situ hybridization (FISH).

### Fluorescence in situ hybridization (FISH)

FISH of whole scaphognathite tissues from an individual not used for the preparation of the clone libraries was exactly carried out as previously described [24]. We used a probe with 0 mismatches to the gammaproteobacterial symbiont sequence S2-12 (Table 1), Rexogam1268RT (5′-CTTTCTGGGATTRGCTTGCTCT-3′) [24], a probe with 0 mismatches to the epsilonproteobacterial symbiont sequences S1-4 (Table 1), RexoepsTG996 (5′-CTGTCGGATTCTCTCAAT-3′) [24], and the general deltaproteobacterial probe SRB385 [36]. Sections were mounted in a mixture of Citifluor and Vectashield and examined using a fluorescence microscope (Zeiss Axioskop, Germany).

### DNA extraction and PCR amplification

Genomic DNA was extracted from four shrimp individuals. The shrimp were dissected under sterile conditions and DNA from the epibiotic community on the branchiostegites and/or on the modified mouthparts was isolated using the “PowerSoil DNA extraction kit” (MO BIO Laboratories, Carlsbad, CA, USA) according to the provided protocol. Bacterial 16S rRNA gene fragments were PCR-amplified in eight parallels in a 20-cycle PCR at an annealing temperature of 50°C with the general bacterial primer set 8F and 1492R [59]. Fragments of ATP citrate lyase genes (*aclA* and *aclB*) were amplified using the primer sets F2/R5 (*aclA*) and 892F/1204R (*aclB*) [39], [60]. For the amplification of fragments of the genes coding for the large subunit of RubisCO form I and II, the primer sets *cbbL*F/*cbbL*R and *cbbM*F/*cbbM*R were used [61]. Fragments of the *soxB* and *aprA* gene were amplified using the primer sets *soxB*432F/*soxB*1446B and *aps*1F/*aps*4R, respectively [44], [62]. Fragments of the *hynL* gene were amplified with the primer set *hynS*330F/*hynL*419R [41]. For each functional gene fragment five parallel reactions were amplified and subsequently pooled. Bacterial 16S rRNA genes were amplified from DNA of all 4 individuals, while functional genes were amplified from individual 4 only. In this case, 16S rRNA as well as functional gene fragments were amplified separately from DNA extracted from branchiostegites and mouth parts.

### Cloning and sequencing

The pooled amplified PCR products were gel-purified using the QIAGEN QIAquick gel extraction kit (Qiagen, Hilden, Germany) and cloned into pCR4-TOPO plasmid vectors with the TOPO-TA cloning kit (Invitrogen, Carlsbad, CA, USA) as described by the manufacturer. An environmental clone library for each gene was constructed (Table S1). Colonies were picked and analyzed for the insert-containing plasmid by direct PCR with the vector primers M13F and M13R followed by gel electrophoreses of the amplified products. PCR products of the correct size were sequenced using the M13 primer set. Sequencing was performed using the BigDye Terminator v1.1 sequencing kit in a 3730xl DNA Analyzer (Applied Biosystems, Carlsbad, CA, USA) as specified by the manufacturer.

### Phylogenetic analysis

All sequences were edited with ChromasPro c.c1.33 and compared to the NCBI database using BLAST [63]. Operational taxonomic units (OTUs) were defined based on 99% nucleotide sequence identity for the 16S rRNA gene sequences, and 98% amino acid sequence identity for functional genes. The 16S rRNA gene sequences were aligned with the ARB software (www.arb-home.de) using the ARB FastAligner utility [64]. The sequence alignment was manually adjusted based on known secondary structures. Sequences of functional genes were aligned using Clustal X [65] and manually adjusted using BioEdit [66]. Maximum-Likelihood based trees and 100 bootstrap replicates were constructed using PhyML [67]. In order to verify the tree topology, aligned sequences were imported into PAUP (Version 4.0b10, Sinauer Associates, Sunderland, MA, USA) for further phylogenetic analyses using Neighbour-Joining and Maximum-Parsimony algorithms (for details see [39]).

### Nucleotide sequence accession numbers

The sequence data have been submitted to EMBL/GenBank/DDBJ databases under accession numbers FN658693-FN658702, FN659777-FN659803 and FN661670.

## Supporting Information

Table S1 Functional gene clone library results.
(PDF)

Figure S1 Phylogenetic tree based on translated *aclB* sequences. The tree was calculated using the Neighbor-Joining method. Bootstrap values are shown as percentages of 1000 bootstrap replicates. Sequences obtained in this study are depicted in red. Scale bar represents 5% estimated sequence divergence.
(TIF)

Figure S2 Phylogenetic tree based on translated *cbbM* sequences. The tree was calculated using the Neighbor-Joining method. Bootstrap values are shown as percentages of 1000 bootstrap replicates. Sequences obtained in this study are shown in yellow. Scale bar represents 10% estimated sequence divergence.
(TIF)

Figure S3 Phylogenetic tree based on translated *hynL* sequences. The tree was calculated using the Maximum-Likelihood method. Bootstrap values are shown as percentages of 100 bootstrap replicates. Sequences obtained in this study are highlighted with colors. Scale bar represents 20% estimated sequence divergence.
(TIF)
