# Supplementary material for: Pathways of Carbon and Energy Metabolism of the Epibiotic Community Associated with the Deep-Sea Hydrothermal Vent Shrimp Rimicaris exoculata
Source: PLoS One. 2011 Jan 7;6(1):e16018. doi: 10.1371/journal.pone.0016018 (PMC3017555; doi:10.1371/journal.pone.0016018)
Supplement: Table S1 — Functional gene clone library results. (PDF) [file pone.0016018.s001.pdf]

Pathways of carbon and energy metabolism of the epibiotic community associated with the vent shrimp *Rimicaris exoculata*

Supplementary Table

Table S1. Functional gene clone library results.

Individual No.	4	4
Sampled body part	BS	MP
aclAE6	4	7
aclAA4	3	-
aclAD3	1	-
aclAF12	-	6
aclAG7	-	5
aclAB2	-	2
aclBA11	11	11
aclBD6	7	1
aclBA4	1	-
aclBB11	1	-
aclBD9	1	-
aclBA3	1	-
aclBC7	1	-
aclBC5	-	6
aclBB7	-	2
aclBB10	-	10
aclBB3	-	1
cbbM1	-	10
soxBE12	58	24
soxBA1	4	2
soxBC1	5	-
aprH8 (delta)	31	8
aprF5 (delta)	1	-
aprA9 (gamma)	6	27
aprD3 (gamma)	2	1
hydC12 (epsilon)	27	25
hydG9 (delta)	16	10
hydA12 (epsilon)	-	1
