# Supplementary figures and images for: Pathways of Carbon and Energy Metabolism of the Epibiotic Community Associated with the Deep-Sea Hydrothermal Vent Shrimp Rimicaris exoculata
Source: PLoS One. 2011 Jan 7;6(1):e16018. doi: 10.1371/journal.pone.0016018 (PMC3017555; doi:10.1371/journal.pone.0016018)

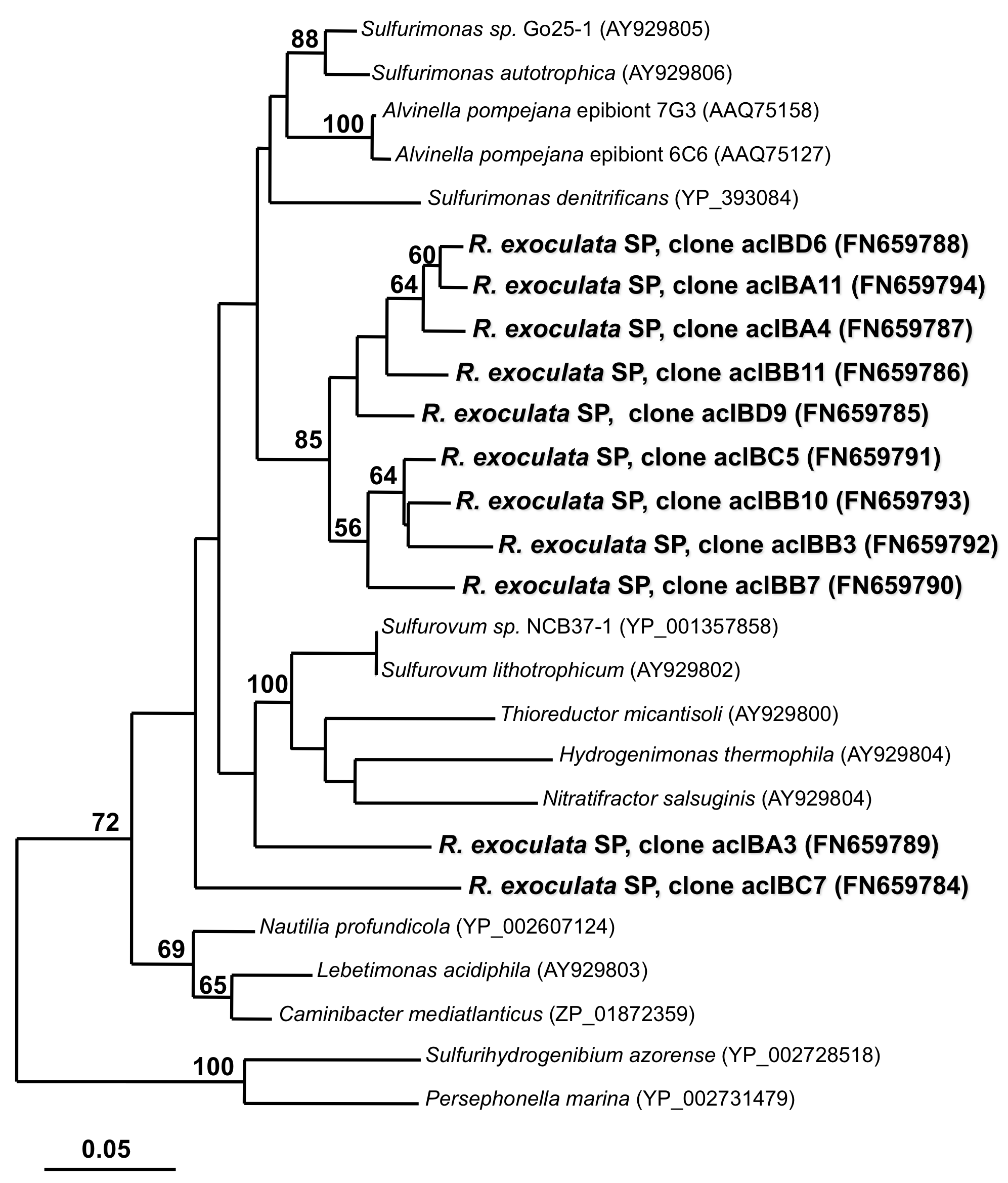

Supplement: Figure S1 — Phylogenetic tree based on translated aclB sequences. The tree was calculated using the Neighbor-Joining method. Bootstrap values are shown as percentages of 1000 bootstrap replicates. Sequences obtained in this study are depicted in red. Scale bar represents 5% estimated sequence divergence. (TIF) [file pone.0016018.s002.tif]

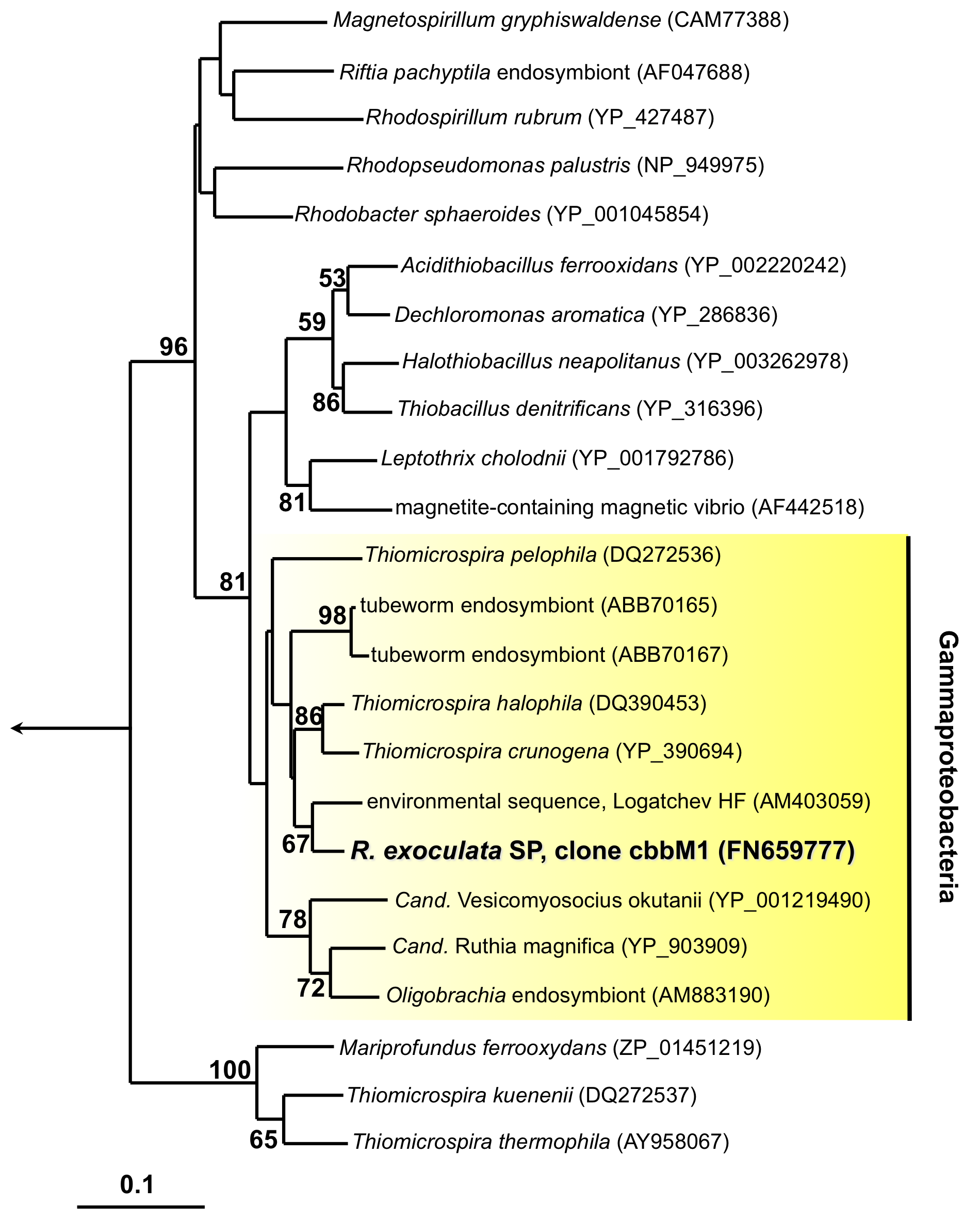

Supplement: Figure S2 — Phylogenetic tree based on translated cbbM sequences. The tree was calculated using the Neighbor-Joining method. Bootstrap values are shown as percentages of 1000 bootstrap replicates. Sequences obtained in this study are shown in yellow. Scale bar represents 10% estimated sequence divergence. (TIF) [file pone.0016018.s003.tif]

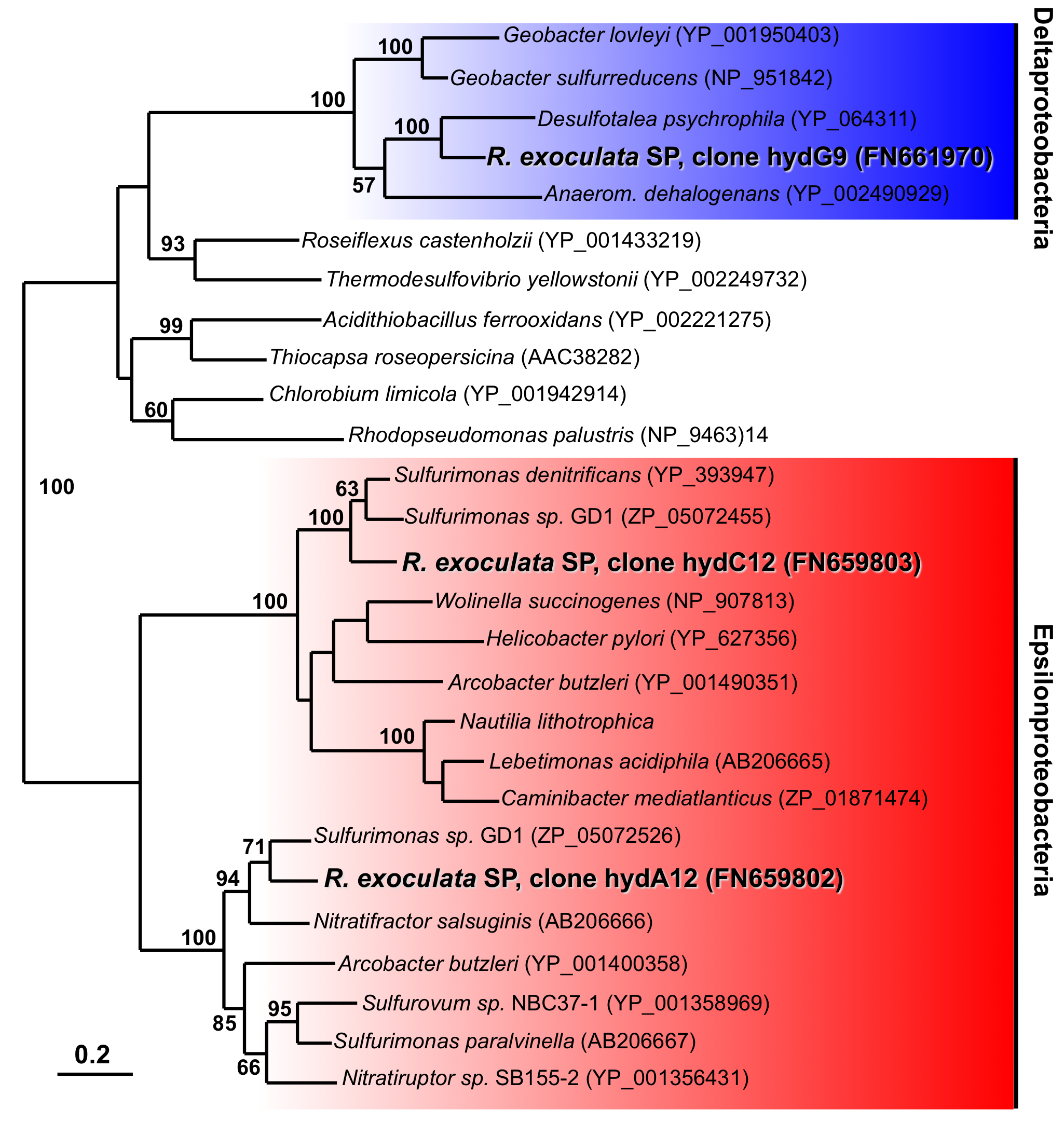

Supplement: Figure S3 — Phylogenetic tree based on translated hynL sequences. The tree was calculated using the Maximum-Likelihood method. Bootstrap values are shown as percentages of 100 bootstrap replicates. Sequences obtained in this study are highlighted with colors. Scale bar represents 20% estimated sequence divergence. (TIF) [file pone.0016018.s004.tif]
